# Behavioral Predictors of Intention to Use a Text Messaging Reminder System Among People Living With HIV in Rural Uganda: Survey Study

**DOI:** 10.2196/42952

**Published:** 2023-05-05

**Authors:** Jeffrey I Campbell, Isaac Aturinda, Evans Mwesigwa, Annabella Habinka, Michael Kanyesigye, Richard J Holden, Mark J Siedner, John D Kraemer

**Affiliations:** 1 Boston Medical Center Boston, MA United States; 2 Mbarara University of Science and Technology Mbarara Uganda; 3 Makerere University Kampala Uganda; 4 School of Public Health Indiana University Bloomington, IN United States; 5 Massachusetts General Hospital Massachusetts, MA United States; 6 Department of Health Management and Policy Georgetown University School of Health Washington, DC United States

**Keywords:** mobile health, mHealth, HIV, intention, SMS, cellular phone, cell phone, Africa, reminder, alert, notification, prompt

## Abstract

**Background:**

The expansion of cellular phones in sub-Saharan Africa spurred the development of SMS text message–based mobile health (mHealth) technology. Numerous SMS text message–based interventions have attempted to increase retention in care for people living with HIV in sub-Saharan Africa. Many of these interventions have failed to scale. Understanding theory-grounded factors leading to mHealth acceptability is needed to create scalable, contextually appropriate, and user-focused interventions to improve longitudinal HIV care for people living with HIV in sub-Saharan Africa.

**Objective:**

In this study, we aimed to understand the relationship between constructs from the Unified Theory of Acceptance and Use of Technology (UTAUT), constructs identified in previous qualitative research, and behavioral intention to use a novel SMS text message–based mHealth intervention designed to improve care retention among people living with HIV initiating treatment in rural Uganda.

**Methods:**

We conducted a survey of people living with HIV who were newly initiating HIV care in Mbarara, Uganda, and had agreed to use a novel SMS text message–based system that notified them of abnormal laboratory results and reminded them to return to the clinic. Survey items assessed behavioral intention to use the SMS text messaging system; constructs from UTAUT; and demographics, literacy, SMS text messaging experience, HIV status disclosure, and social support. We used factor analysis and logistic regression to estimate the relationships between UTAUT constructs and the behavioral intention to use the SMS text messaging system.

**Results:**

A total of 249 participants completed the surveys, of whom 115 (46.2%) expressed high behavioral intention to use the SMS text messaging intervention. In a multivariable analysis, we found that performance expectancy (adjusted odds ratio [aOR] of the scaled factor score 5.69, 95% CI 2.64-12.25; *P*<.001), effort expectancy (aOR of the scaled factor score 4.87, 95% CI 1.75-13.51; *P*=.002), and social influence (measured as a 1-unit Likert score increase in the perception that clinical staff have been helpful in the use of the SMS text messaging program; aOR 3.03, 95% CI 1.21-7.54; *P*=.02) were significantly associated with high behavioral intention to use the SMS text messaging program. SMS text messaging experience (aOR/1-unit increase 1.48, 95% CI 1.11-1.96; *P*=.008) and age (aOR/1-year increase 1.07, 95% CI 1.03-1.13; *P*=.003) were also significantly associated with increased odds of high intention to use the system.

**Conclusions:**

Performance expectancy, effort expectancy, and social influence, as well as age and SMS experience, were drivers of high behavioral intention to use an SMS text messaging reminder system among people living with HIV initiating treatment in rural Uganda. These findings highlight salient factors associated with SMS intervention acceptability in this population and indicate attributes that are likely to be key to the successful development and scaling of novel mHealth interventions.

## Introduction

### Background

In 2020, there were an estimated 93 cellular phone subscriptions per 100 people living in sub-Saharan Africa [1]. Numerous interventions have attempted to leverage this widespread cellular phone coverage to address health gaps in the region, especially with respect to people living with HIV [2,3]. Retention in HIV care is complex, and barriers to long-term engagement range from individuals’ physical and mental health to resource constraints and social and cultural factors such as stigma [4]. The ubiquity of cellular phones makes mobile health (mHealth) interventions a promising tool to overcome many of these challenges to retention in care. A recent meta-analysis found that mobile phone reminders significantly improved retention in care for people living with HIV [5]. However, despite promising pilot studies, many mHealth interventions have not been scaled in sub-Saharan Africa [6,7]. Barriers such as unstable funding, unreliability of technology, and health systems’ lack of capacity to integrate electronic data can impede the broad uptake of mHealth interventions [7]. Although many studies have examined the acceptance and acceptability during intensive pilot intervention periods, there are fewer data that capture acceptability after the pilot phase.

A postpilot, theory-grounded understanding of mHealth acceptability and use among people living with HIV in sub-Saharan Africa is critical for creating sustainable, contextually adapted, user-focused interventions to improve longitudinal HIV-related care [8]. Models that integrate social and cultural contexts with perceptions of technology utility are particularly valuable to understand mHealth uptake among people living with HIV in sub-Saharan Africa, where previous studies have found that social support and influence are among the key perceived benefits of mHealth technologies [9]. Theoretical models of technology acceptance have been developed in high-resource settings to explain the links between user perceptions, social influences, intentions to use, and the actual use of technologies [10-14]. These models have also been applied to health technologies for HIV care in resource-limited settings [9,14-16]. Central to many of these models is *behavioral intention*, a concept that reflects prospective users’ perceived intention to use a new technology. Behavioral intention is often used as a measure of acceptance in technology acceptance models because it can be more readily measured than the actual use of many technologies and generally correlates with technology use.

In a previous qualitative study of people living with HIV in rural Uganda, we examined user attitudes toward an SMS text message notification sent to alert patients with low CD4 counts and to recall them to the clinic to initiate antiretroviral therapy (ART). On the basis of this investigation, we developed the Technology Acceptance Model for Resource-Limited Settings [17]. This model situates intended health outcomes within a framework of the behavioral factors leading to technology use and the downstream intervening factors that attenuate or propel the link between technology use and health outcomes. The qualitative study provided insights into the links between technology acceptance and anticipated health outcomes. However, as a qualitative study, it did not estimate the relationships between established technology acceptance constructs and behavioral intention to use an mHealth technology.

### Objective

Here, we attempted to test the above conceptual framework by examining factors from technology acceptance theory—specifically, from the Unified Theory of Acceptance and Use of Technology (UTAUT) [13], which we hypothesized affects the behavioral intention to use SMS text message–based health interventions among people living with HIV. Our primary objective was to quantitatively estimate the relationships between these behavioral constructs and behavioral intention to use an mHealth application among intended end users, specifically people living with HIV in a sub-Saharan African setting.

## Methods

### Population and Setting

We conducted a standardized survey of people living with HIV who initiated HIV care at the Mbarara Regional Referral Hospital Immune Suppression Syndromes Clinic in Mbarara, Uganda. Our study was conducted in conjunction with a clinic-wide rollout of an SMS text message–based reminder intervention. On the basis of the results from a prospective before-and-after clinical trial at the same clinic (NCT01579214) [18], the intervention aimed to improve patient-provider communication, remind individuals of upcoming appointments, and notify them of laboratory results (ie, CD4 count and viral load results). ART-naïve people living with HIV who were >18 years old and were initiating care at the clinic were offered voluntary enrollment in the SMS text messaging reminder program by clinic staff on the day of ART initiation. In this program, standardized SMS text messages were automatically sent 7 days and 1 day before the scheduled clinic return dates. The messages were sent in the morning. Participants could choose to receive messages in the region’s most common languages: English, Runyankole, or Luganda. The messaging application was developed by a local technology company (iStreams). It operated with a modem that interacted with the clinic’s server to send messages through a phone network (Airtel). The SMS text message contained a reminder instructing patients to return to the clinic for their appointments. On the basis of the results of a previous trial that showed decreased SMS text message uptake owing to encoded or password-protected messages [19], messages were not encoded or password protected. To avoid loss of confidentiality or disclosure, the messages did not contain patient names, nor mentioned HIV or AIDS.

### Survey Design and Variable Selection

On the day of their clinic intake, we invited the first 2 to 3 patients each day who were enrolled in the SMS text messaging reminder program to participate in this survey. After providing informed consent, participants were invited to complete a detailed survey. The survey could be completed either as a self-administered written questionnaire or as an interviewer-administered verbal questionnaire per participants’ preference. The survey was available in Runyankole, the predominant language in Mbarara. A trained research assistant administered the surveys on the day of clinic enrollment. The survey contained questions about demographics (sex, age, location of housing, literacy, and education), cell phone use, HIV-related stigma, HIV disclosure, available social support, and survey measures of constructs from the UTAUT model [13]. Survey items measuring UTAUT constructs have been validated in other contexts [13] and were adapted for our study (Table 1). Notably, although self-efficacy, attitudes, and anxiety are not included in the original UTAUT model [13], we measured these constructs in our setting, given our application of this model in a new context, as has been used in other “extended” UTAUT models for health services [20]. All survey items were measured on a 5-point Likert scale (ranging from “strongly disagree” to “strongly agree”). Of note, because the survey was obtained at the time of SMS text messaging program enrollment, we sought to measure the perceived “acceptability” of the program, rather than use-based “acceptance.”

On the basis of previous qualitative research on SMS text messaging acceptability in this context [17], we measured the following constructs:

1. Demographics and HIV disease status

Participants reported their age, gender, and location of their homes during the survey. When available, the CD4 count at the time of study enrollment was retrieved from clinical records.

2. Literacy and educational attainment

To assess literacy, we asked the participants to read a short sentence in Runyankole or English according to their preferences. Participants were deemed literate if they could read all or parts of the sentence in their preferred language. Participants were asked about the highest level of education they had completed.

3. SMS text message experience

Because the ability to send an SMS text message was believed to represent cell phone familiarity, we defined texting experience as an ordered categorical variable based on the number of reported SMS text messages sent per week. We inspected a Lowess plot and found that the relationship between texting exposure and behavioral intention was logit-linear across categories. We therefore included this variable as a continuous variable in models.

4. HIV status disclosure

Previous HIV status disclosure was measured using a single binary variable that represented whether the participants had disclosed their HIV diagnosis to anyone.

5. Social support

We measured social support using a validated social support scale [21]. This scale uses 10 Likert-scale questions about family and community social support, which are averaged and dichotomized into “high social support” and “low social support” categories.

**Table 1:** Unified Theory of Acceptance and Use of Technology constructs and survey items.

English		Runyankole	Comments
**PE^a^—degree to which the SMS system will help or be useful for patients to receive care**			
	PE1. I would find the SMS program useful.	Nka nshangire enkora y’okusindika obutumwa bwesiimu obuhandikirwe eri ey’omugasho.	—^b^
	PE2. Using the SMS program enables me to get care from the clinic more quickly.	Okukozesa enkora y’okusindika obutumwa bwesiimu obuhandikirwe nikimpweera kurahuka kutunga obujanjabi aha kirinika.	—
	PE3. Using the SMS program increases my ability to get help from the clinic.	Okukozesa enkora y’okusindika obutumwa bwesiimu obuhandikirwe nikwongyera aha kubaasakutunga obujanjabi aha kirinika.	—
	PE4. If I use the SMS program, I will increase my chances of getting help at the clinic.	Nabankorise enkora y’okusindika obutumwa bwesiimu obuhandikirwe, ninyija kwongyera aha migisha yangye eyokutunga obujanjabi aha kirinika.	Dropped owing to undefined estimate in factor analysis (high correlation with item PE3).
**EE^c^—degree to which the SMS system is easy to use**			
	EE1. My interaction with the SMS program would be clear and understandable.	Okukoresa enkora y’okusindika obutumwa bwesiimu obuhandikirwe kukabaire kurikushoboroka kandi kurikwetegyerezibwa.	—
	EE2. It would be easy for me to become skillful at using the SMS program.	Kikanyoroobeire okutunga obukugugu omukukoresa enkora y’okusindika obutumwa bwesimu obuhandikirwe.	—
	EE3. I would find the SMS program easy to use.	Nka nshangire enkora y’okusindika obutumwa bwesiimu obuhandikirwe eyoroobi kukoresa.	—
	EE4. Learning to operate the SMS program is easy for me.	Okwega kukoresa enkora y’okusindika obutumwa bwesiimu obuhandikirwe nikunyoroobera.	—
**ATT^d^—degree to which patients hold positive or negative perception of using the SMS program**			
	ATT1. Using the SMS program is a good idea.	Nekitekateko kirungi okukoresa enkora y’okusindika obutumwa bwesiimu obuhandikirwe.	—
	ATT2. The SMS program makes work more interesting.	Enkora y’okusindika obutumwa bwesiimu obuhandikirwe nikyongyera omwete kwokukora.	—
	ATT3. Working with the SMS program is fun.	Okukoresa enkora y’okusindika obutumwa bwesiimu obuhandikirwe na mashemererwa gonka.	—
	ATT4. I like working with the SMS program.	Ninkunda kukoresa enkora y`okusindika obutumwa bwesiimu obuhandikirwe.	—
**SI^e^—degree to which patients perceive that other people important to them want them to use the SMS system**			
	SI1. People who influence my behavior think that I should use the SMS program.	Abantu abarikuretera natwaza nkokundikutwaza omumicwe nibatekateka ngu nshemereire kukoresa enkora y’okusindika obutumwa bwesimu obuhandikirwe.	Dropped owing to undefined estimate in factor analysis (high correlation with item SI2).
	SI2. People who are important to me think that I should use the SMS program.	Abantu abunkutwara nkab’omugasho aharinye nibatekateka ngu nshemereire kukoresaenkora y’okusindika obutumwa bwesiimu obuhandikirwe.	Used as a measured variable in multivariable analysis.
	SI3. The clinic staff have been helpful in the use of the SMS program.	Abakozi ba kirinika babeire bari abahwezi omukukoresa enkora y’okusindika obutumwa bwesiimu obuhandikirwe.	Used as a measured variable in multivariable analysis.
	SI4. In general, the clinic has supported the use of the SMS program.	Okutwariza hamwe, kirinika ehagiire enkora y’okusindika obutumwa bwesiimu obuhandikirwe.	Dropped owing to undefined estimate in factor analysis (high correlation with items SI2 and SI3).
**FC^f^—degree to which patients perceive sufficient resources and infrastructure to use the SMS system**			
	FC1. I have the resources necessary to use the SMS program.	Nyiine ebirikwetagisa kukoresa enkora y’okusindika obutumwa bwesiimu obuhandikirwe.	—
	FC2. I have the knowledge necessary to use the SMS program.	Nyine amagezi agarikwetagisa kukoresa enkora y’okusindika obutumwa bwesiimu obuhandikirwe.	—
	FC3. The SMS program is not compatible with other SMS programs I use.	Enkora y’okusindika obutumwa bwesiimu obuhandikirwe terikukwatirana nezindi nkora nkezo ezindikukoresa.	—
	FC4. A specific person (or group) is available for assistance with SMS program difficulties.	Omuntu nari abantu batoraine bariho kumpwera ebizibu byenkora y’okusindika obutumwa bwesiimu obuhandikirwe.	Dropped owing to poor loading in EFA^g^.
**SE^h^—degree to which patients perceive they have the aptitude to use the SMS system**			
	I could successfully get the information I need using the SMS program...	Nimbaasa kutunga amakuru goona agindikwenda ndikwejunisa enkora y’okusindika obutumwa bwesiimu obuhandikirwe...	—
	SE1. If there was no one around to tell me what to do as I go.	Habahatariho muntu wena kungambira okundatwaze nenkora egi.	—
	SE2. If I could call someone for help if I got stuck.	Kunakuba nimbaasa kugira omuntu owunayeeta naheza kuremererwa.	Dropped owing to poor loading in EFA.
	SE3. If I had a lot of time, I could get the information I needed from the SMS program.	Naba nyine obwire bwingi mbaasa kutunga amakuru agindikwenda omu nkora y`okusindika obutumwa bwesimu obuhandikirwe.	—
	SE4. If I had just the help of the information, I received at the clinic about the SMS program.	Kurinintunga obuyambi bwekyokukora nkobunatungire aha kirinika obukwatiraine nenkora y’okusindika obutumwa bwesimu obuhandikirwe.	—
**Anxiety—degree to which patients feel apprehension or fear about using the SMS program**			
	Anxiety 1. I feel apprehensive about using the SMS program.	Nyine obutagubwagye hamwe nobwooba bwokukoresa enkora y`okusindika obutumwa bwesiimu obuhandikirwe.	—
	Anxiety 2. It scares me to think that I could lose a lot of information using the SMS program by hitting the wrong key.	Nikindetera obwooba okutekateka ngu kunakunyiiga eipesha erigwaire mbaasa kuburwaho amakuru maingi naba ninkukoresa enkora y’okusindika obutumwa bwesiimu obuhandikirwe.	—
	Anxiety 3. I hesitate to use the SMS program for fear of making mistakes I cannot correct.	Tinkurahukiriza kukoresa enkora y’okusindika obutumwa bwesiimu obuhandikirwe aha bwokutiina kukora enshobi enzintarikubaasa kugoroora.	—
	Anxiety 4. The SMS program is somewhat intimidating to me.	Enkora y`okusindika obutumwa bwesiimu obuhandikirwe nentinisamu kakye.	—
**BI^i,j^—degree to which patients intend to use the SMS system**			
	BI1. I intend to use the SMS program in the next 3 months.	Ninyenda kukoresa enkora y’okusindika obutumwa bwesiimu obuhandikirwe omumyezi eshatu erikwaija.	—
	BI2. I predict I would use the SMS program in the next 3 months.	Nintebereza kwija kukoresa enkora y’okusindika obutumwa bwesiimu obuhandikirwe omumyezi eshatu erikwaija.	—
	BI3. I plan to use the SMS program in the next 3 months.	Nintekateka kwija kukozesa enkora y’okusindika obutumwa bwesiimu obuhandikirwe omumyezi eshatu erikwaija.	—

^a^PE: performance expectancy.

^b^Not available.

^c^EE: effort expectancy.

^d^ATT: attitude toward using technology.

^e^SI: social influence.

^f^FC: facilitating condition.

^g^EFA: exploratory factor analysis.

^h^SE: self-efficacy.

^i^BI: behavioral intention to use.

^j^Consolidated into 1 variable, measuring the highest value across questions.

### Statistical Analysis

We used descriptive and summary statistics to characterize the study population. For constructs drawn from the UTAUT model, we performed exploratory factor analysis (EFA) to examine the relationship between the measured survey items and the latent constructs they measured. We elected to perform EFA (rather than confirmatory factor analysis) because we posited adaptations to the overall UTAUT model (eg, adapting questions to our setting; Table 1) and because UTAUT was designed to describe the acceptability of nonhealth technology in well-resourced settings, rather than mHealth in a sub-Saharan African context. We removed items that performed poorly in the EFAs or that yielded undefined estimates in the factor analysis, owing to high correlation with one another (Table 1). We then attempted to perform structural equation modeling to understand the relationship between behavioral intention, UTAUT variables, and other covariates. However, the structural equation models could not be identified based on the number of latent variables, their associations, and the number of available observations. For our primary analysis, we therefore fit logistic regression models in which we represented latent predictor constructs with factor scores derived from separate measurement models for each.

Our primary outcome of interest was behavioral intention to use the SMS text messaging program. Behavioral intention was measured using 3 Likert-scale questions (Table 1). Owing to the low variability in responses and near-perfect concordance between the items, we created a binary outcome variable to characterize “high” behavioral intention (defined as listing “strongly agree” for at least 1 of the 3 behavioral intention questions) and “low” behavioral intention (defined as listing a rating of less than “strongly agree” for all the behavioral intention questions).

Our primary predictors of interest were constructs from the UTAUT model, including (1) performance expectancy, (2) effort expectancy, (3) attitudes toward technology, (4) facilitating conditions, (5) anxiety about technology use, (6) self-efficacy, and (7) social influence. We represented these constructs in the models as factor scores. To create factor scores, we first performed EFA to identify survey items with goemin-rotated loadings of >0.4 [22] for all UTAUT predictor constructs, except for social influence, which was represented as 2 observed variables. The identified survey items were then included in the confirmatory factor analysis to generate factor scores. Factor scores were divided by their IQRs so that a 1-unit increase in the score was equivalent to the difference between the middle of the bottom half and the middle of the top half. Because items measuring social influence were highly correlated, we represented this construct as 2 measured variables (“People who are important to me think that I should use the SMS program” and “The clinic staff have been helpful in the use of the SMS program”) instead of as factor scores in the models. These questions were selected because they were found to have the highest face validity among questions measuring social influence. In addition, we removed one item (“If I use the SMS program, I will increase my chances of getting help at the clinic”) from the measurement of performance expectancy owing to its high correlation with another performance expectancy item in the confirmatory factor analysis.

We first constructed a univariable logistic regression to evaluate the associations between UTAUT constructs and covariates with behavioral intention. We then conducted a multivariable logistic regression to identify the correlates of high (vs low) behavioral intention to use the SMS text messaging system. Owing to a priori hypotheses about their effect on behavioral intention, all covariates were included in the multivariable model. Owing to collinearity between the UTAUT constructs’ factor scores, we used a forward selection strategy to identify UTAUT constructs that were independent significant predictors of high versus low behavioral intention, using *P*<.20 for inclusion in the final model. The significance in the final model was set at *P*<.05. Patients with missing survey data (n=3) were excluded from the multivariable analysis. Patients were only excluded from specific univariable tests and factor analysis when the items considered in the specific test or analysis were missing. Because odds ratios are liable to be misinterpreted when the outcome of interest is not rare, we calculated and graphed associations as adjusted differences in the probability of the outcome following regression. We did this by using the average marginal effects with other covariates held at the observed levels.

We conducted 3 sensitivity analyses with multivariable logistic regression models using low versus high behavioral intention as the outcome and different variable inclusion strategies to select UTAUT constructs and covariates. First, we constructed a multivariable model that included all UTAUT constructs, with age and sex as covariates. Second, we created a model that included all UTAUT constructs and all covariates. Finally, we constructed models that included each UTAUT construct separately plus all covariates.

Analyses were conducted using Stata (version 17; StataCorp) and Mplus (version 8; Muthén & Muthén), which was run within Stata via the *runmplus* suite of commands.

### Ethics Approval

The study was reviewed and approved by the Ethics Review Committee of the Mbarara University of Science and Technology (13/10-15), the Institutional Review Board of Massachusetts General Hospital (2015P002572), and the Uganda National Council for Science and Technology (SS 4008).

## Results

### Participant Characteristics

A total of 249 participants were enrolled in the survey study and completed the surveys. The mean age was 30.6 (SD 9.2) years, and 56.2% (140/249) of the patients were female. The median CD4 count at the time of enrollment was 311 (IQR 145-524). Of the 249 participants, 226 (90.8%) participants were literate and 219 (88%) endorsed high social support. Only 57.4% (143/249) of the participants noted that they had disclosed their HIV status. The majority (155/249, 62.2%) had sent fewer than 3 SMS text messages during the preceding week. Multimedia Appendix 1 summarizes the factor loadings for the survey items measuring the UTAUT constructs.

### Predictors of High (vs Low) Behavioral Intention

In the univariable analysis, literacy, number of SMS text messages sent in the preceding week, and social support were significantly associated with intention to use the SMS text messaging program (Table 2). The mean scaled factor scores and measured survey responses (for the 2 social influence variables) differed significantly between participants with low and high behavioral intention (Table 2).

**Table 2:** Univariable and multivariable analysis of predictors of behavioral intention.

				Low Intention (n=134)		High Intention (n=115)		Univariable analysis					Multivariable analysis		
								OR^a^ (95% CI)		*P* value		aOR^b^ (95% CI)^c^			*P* value
**Patient characteristics**															
	Age (years), mean (SD)		29.8 (8.6)		31.6 (9.8)		1.02 (0.99-1.05)		.12		1.07 (1.03-1.13)			*.003* ^d^	
	**Sex, n (%)**														
		Male	55 (41)		54 (47)		Ref^e^		Ref		Ref			Ref	
		Female	79 (59)		61 (53)		0.79 (0.46-1.30)		.35		1.62 (0.73-3.57)			.24	
	**Literate, n (%)**														
		No	19 (14)		4 (3)		Ref		Ref		Ref			Ref	
		Yes	115 (86)		111 (97)		4.58 (1.51-13.90)		*.007*		0.18 (0.03-1.18)			.07	
	**SMS sent in the past week (per 1-unit increase in the ordinal scale), n (%)**							1.79 (1.48-2.18)		*<.001*		1.48 (1.11-1.96)			*.008*
		None	89 (67)		35 (30)										
		1-2	16 (12)		15 (13)										
		3-5	12 (9)		16 (14)										
		6-10	12 (9)		23 (20)										
		11-20	2 (2)		19 (17)										
		>20	2 (2)		7 (6)										
	**High social support, n (%)**														
		No	22 (16)		8 (7)		Ref		Ref		Ref			Ref	
		Yes	112 (84)		107 (93)		2.62 (1.12-6.16)		*.03*		1.06 (0.33-3.44)			.92	
	**History of prior disclosure, n (%)**														
		No	51 (38)		55 (48)		Ref		Ref		Ref			Ref	
		Yes	83 (62)		60 (52)		0.67 (0.40-1.11)		.12		0.47 (0.21-1.02)			.06	
**UTAUT^f^ constructs**															
	Performance expectancy, mean scaled factor score		−0.41		0.51		11.60 (6.39-21.03)^g^		*<.001*		5.69 (2.64-12.25)^g^			*<.001*	
	Effort expectancy, mean scaled factor score		−0.47		0.56		12.32 (6.37-23.81)^g^		*<.001*		4.87 (1.75-13.51)^g^			*.002*	
	Attitudes**,** mean scaled factor score		−0.27		0.31		9.93 (5.45-18.11)^g^		*<.001*		N/A^h^			N/A	
	Facilitating conditions, mean scaled factor score		−0.22		0.25		10.77 (5.72-20.28)^g^		*<.001*		N/A			N/A	
	Anxiety, mean scaled factor score		0.33		−0.38		0.23 (0.13-0.35)^g^		*<.001*		N/A			N/A	
	Self-efficacy, mean scaled factor score		−0.42		0.51		7.61 (4.37-13.26)^g^		*<.001*		N/A			N/A	
	Social influence (“The clinic staff have been helpful in the use of the SMS program”), mean Likert response		3.11		3.34		3.59 (1.92-6.71)^i^		*<.001*		3.03 (1.21-7.54)^i^			*.02*	
	Social influence (“People who are important to me think that I should use the SMS program”), mean Likert response		2.08		2.83		2.60 (1.87-3.61)^i^		*<.001*		N/A			N/A	

^a^OR: odds ratio.

^b^aOR: adjusted odds ratio.

^c^Adjusted for age, sex, literacy, SMS experience, social support, disclosure status, performance expectancy (normalized factor score), effort expectancy (normalized factor score), and social influence (Likert-scale question).

^d^Values in italics represent significant *P* value.

^e^Ref: reference.

^f^UTAUT: Unified Theory of Acceptance and Use of Technology.

^g^Odds ratio associated with a 1-unit change in the factor score interquartile interval.

^h^N/A: not applicable; excluded from the multivariable model during forward variable selection.

^i^Odds ratio associated with a 1-unit change in the Likert scale.

**Figure 1:**
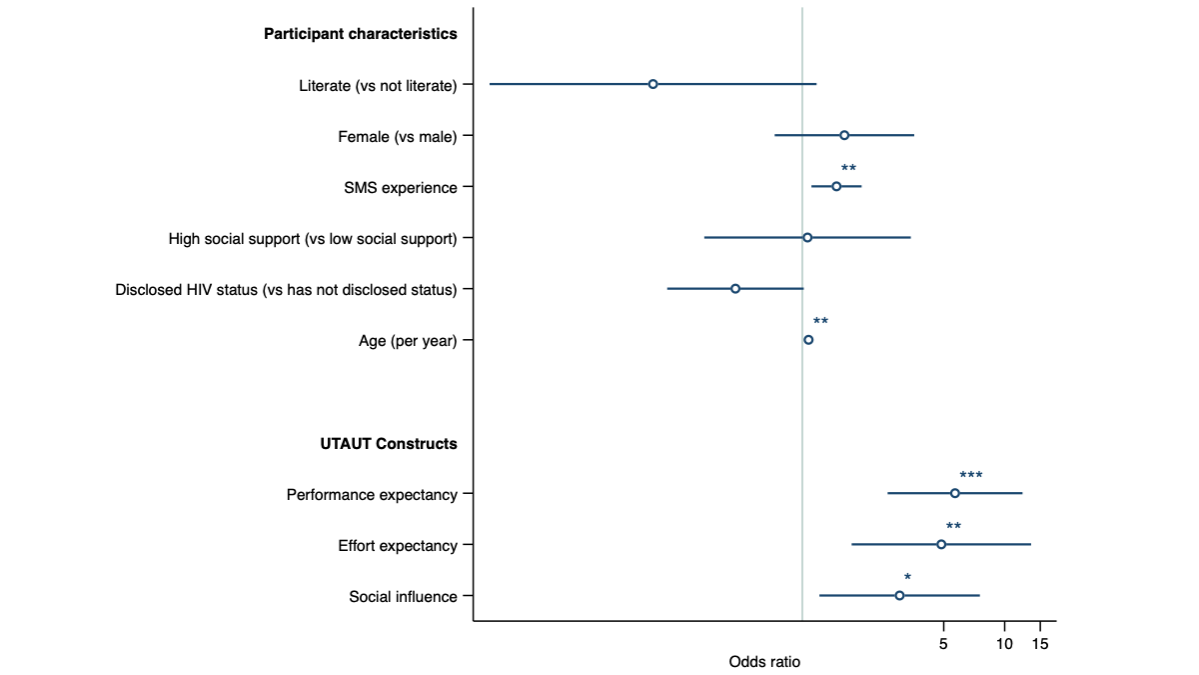
Association of covariates (literacy, sex, SMS experience, social support, disclosure status, and age) and Unified Theory of Acceptance and Use of Technology (UTAUT) constructs on odds of high intention to use the SMS text messaging intervention. All hypothesized non-UTAUT covariates were included in the model. UTAUT constructs were included through a forward selection strategy. *P<.05; **P<.01; ***P<.001.

A total of 1.2% (3/249) of the patients were excluded from the multivariable analysis owing to missing data. In the multivariable analysis, we found that performance expectancy (adjusted odds ratio [aOR] 5.69, 95% CI 2.64-12.25; *P*<.001); effort expectancy (aOR 4.87, 95% CI 1.75-13.51; *P*=.002); and social influence (measured as the perception that clinical staff have been helpful in the use of the SMS text messaging program; aOR 3.03, 95% CI 1.21-7.54; *P*=.02) were significantly associated with a high behavioral intention to use the SMS text messaging program (Figure 1; Table 2). Texting experience (aOR/1-unit increase 1.48, 95% CI 1.11-1.96; *P*=.008) and age (aOR/1-year increase 1.07, 95% CI 1.03-1.13; *P*=.003) were also significantly associated with increased odds of high intention. Multimedia Appendix 2 demonstrates the marginal effects of social influence, performance expectancy, and effort expectancy on the probability of high behavioral intention. Multimedia Appendix 3 shows the marginal effects of covariates on the probability of high behavioral intention.

### Sensitivity Analyses

Sensitivity analyses using different strategies to select covariates for multivariable models yielded results similar to those of the main model. First, in a model that included UTAUT constructs and the covariates age and sex, performance expectancy (aOR 6.40, 95% CI 2.80-14.66; *P*<.001) and social influence (measured as the perception that “The clinic staff have been helpful in the use of the SMS program”; aOR 2.53, 95% CI 1.03-6.24; *P*=.04) remained significantly associated with behavioral intention (Multimedia Appendix 4). Second, in a model that included all UTAUT constructs and all covariates, performance expectancy (aOR 6.78, 95% CI 2.91-15.80; *P*<.001) and social influence (measured as the perception that “The clinic staff have been helpful in the use of the SMS program”; aOR 3.20, 95% CI 1.23-8.38; *P*=.02) were again significantly associated with behavioral intention (Multimedia Appendix 4). In both sensitivity models, there was evidence of collinearity between the UTAUT constructs, with some variables demonstrating inflated CIs. Finally, when each UTAUT construct was considered in models adjusting for covariates, but no other UTAUT constructs were considered, we found that each construct to be significantly associated with behavioral intention (Multimedia Appendix 4).

## Discussion

### Principal Findings

We found that performance expectancy, effort expectancy, and social influence were significantly associated with high behavioral intention to use an SMS text message–clinic return reminder system among people living with HIV initiating ART in rural Uganda. Although participants were surveyed at the time of texting program initiation, these findings suggest that texting programs that are perceived as useful, low effort, and socially supported or promoted are likely to be most acceptable and engaging to this target population. These results add important behavioral acceptability data to the field of mHealth technology acceptance and acceptability in the region. The significance of performance expectancy and effort expectancy in our study suggests that this study population values technology that is useful and easy to use when estimating their intention to use a new mHealth technology. In addition, the significance of social influence—specifically, a question regarding clinic staff support for use of the SMS text messaging system—suggests the importance of positive social norms, particularly positive impressions from health care team members, in motivating the use of mHealth-based services in this setting.

The UTAUT model has been used and adapted in various resource-limited settings to understand the intention to use new health technologies [23]. Our finding that performance expectancy, effort expectancy, and social influence were significantly associated with behavioral intention to use an SMS text messaging system in Uganda aligns with previous research on mHealth interventions in similar settings. A recent survey and qualitative literature on mobile phone–based health interventions in Africa illustrated the differential importance of the UTAUT constructs. A path analysis of UTAUT constructs examined attitudes toward a mobile interactive voice response system for monitoring childhood illness in Ghana and found that performance expectancy, effort expectancy, and social influence were positively associated with behavioral intention to use the technology [24]. Similarly, a real-world mixed methods acceptability study of an SMS text message–based adherence-monitoring intervention in Uganda concluded that performance expectancy was the key driver of the acceptability of the system [9]. Analogously, qualitative studies from Uganda have found that appealing aspects of mHealth interventions for long-term HIV care include their ability to overcome forgetfulness and stigma, whereas technical issues that could make these interventions more effortful to use have been highlighted as areas for improvement [25,26].

In contrast, in our study, attitudes toward SMS text messaging technology, facilitating conditions, anxiety, and self-efficacy were not significantly associated with behavioral intention to use the technology in a model that also included performance expectancy, effort expectancy, social influence, and covariates. In the original UTAUT model, attitudes, anxiety, and self-efficacy were not found to significantly influence behavioral intention in models that included effort expectancy (which subsumed anxiety, self-efficacy, and attitudes) and performance expectancy (which subsumed attitudes) [13], although these factors were included in subsequent extended UTAUT models for resource-limited settings [20]. Although significant in our models in which attitudes, anxiety, and self-efficacy were the sole UTAUT constructs, our findings suggest that these constructs do not explain behavioral intention in our setting beyond performance expectancy, effort expectancy, and social influence in our population and context. In addition, the lack of significance of attitudes, anxiety, and self-efficacy in the multivariable model could have been due to the relative simplicity of the SMS text messaging system and the penetration of SMS text messages into society (ie, 180/249, 72.3% of respondents reported knowing how to send an SMS). Finally, this finding may also be related to the fact that patients were surveyed before using the SMS text messaging system, making these questions hypothetical for the respondents. Given the challenges with scaling mHealth interventions and the field’s reliance on cross-sectional data to understand acceptability, longitudinal studies of how factors affect their actual use in practice will be valuable to better understand the realized impact of mHealth interventions. For example, perceived self-efficacy or anxiety about technology may become more salient, whereas the importance of effort expectancy may wane over time as people living with HIV become more familiar and versatile with a new technology. In related research, comfort with mHealth tools was found to increase over time among individuals with tuberculosis in high-resource settings [27].

We controlled for several participant characteristics hypothesized to affect the intention to use the SMS text messaging intervention. We found that increasing age was associated with higher behavioral intention. This finding was unexpected; we initially hypothesized that younger people living with HIV would be more comfortable with SMS text messaging technology in general and hence would be more likely to have high behavioral intention. By contrast, younger individuals may have expected more sophisticated technology than a simple SMS text message. Notably, in the pilot study preceding our study, increasing age was associated with decreased time to return to the clinic after an abnormal CD4 result, suggesting that increasing age may be independently associated with the propensity to engage in care [18]. Increased SMS text messaging experience may have offset anxiety about using the SMS text messaging intervention. Most participants in our study had relatively low exposure to SMS text messages (155/249, 62.2% had sent fewer than 3 SMS text messages per week). Our results suggest that as cell phones become more common and SMS text messaging becomes less expensive in resource-limited settings, SMS text message–based interventions may become more acceptable.

Our findings are generally consistent with our previous qualitatively derived frameworks for mHealth acceptability in resource-limited settings [17]. In that analysis, we interviewed people living with HIV who had used the SMS text messaging system to facilitate their return to the clinic. Participants identified factors affecting actual technology use and downstream mediators of the target health outcome (return to the clinic). Perceived ease of use and perceived usefulness were found to be upstream promoters of technology use, which was also affected by confidentiality and disclosure considerations. In this study, the findings that performance expectancy and effort expectancy predicted high behavioral intention corroborate our previous qualitative findings that perceived ease of use and perceived usefulness promote technology use. The UTAUT constructs of performance expectancy and effort expectancy are founded in part upon the concepts of perceived usefulness and perceived ease of use, respectively, as delineated in the technology acceptance model [13], which in turn anchored our Technology Acceptance Model for Resource-Limited Settings framework. Although a history of prior disclosure was not significantly associated with high behavioral intention in our study, our survey study was unlikely to be able to capture the complex relationship between HIV-directed mHealth technology adoption and concerns about disclosure and stigma that have been captured in qualitative research from this setting [17,28].

### Strengths and Limitations

The strengths of this study include the large sample size, enrollment of people living with HIV initiating ART (thus providing a unique insight into the attitudes of this important population), the use of existing technology acceptance theory, and the ability to compare this quantitative analysis with our previous qualitative research in the same setting and with a similar patient population. Our study has several limitations. First, data were obtained from a single setting in rural Uganda, and these findings may not be generalizable to other contexts in which mHealth interventions are used. Second, we adapted and translated a UTAUT questionnaire validated in high-resource settings so that it would be understandably relevant to our study population. Given the low variability in responses to behavioral intention questions, we analyzed behavioral intention as a dichotomous variable. Social desirability biases among participants may have resulted in positive responses to behavioral intention questions, which we overcame by dichotomizing “high” versus “not high” behavioral intention. In addition, the nuanced differences in the terminology used in the English behavioral intention questions may not have been translated thoroughly into Runyankole, limiting variability in responses. Given our modification of the questionnaire and the analysis of the behavioral intention variable, our findings should be considered exploratory. Third, despite our attempts to adapt survey questions to our population and setting, some survey questions did not function well in our analysis, and low variability in responses led to the inability to perform structural equation modeling. Our use of exploratory and confirmatory factor analysis was able to eliminate noncontributory questions.

### Conclusions

In conclusion, we found that performance expectancy, effort expectancy, and social influence were key drivers of high behavioral intention to use an SMS text messaging reminder system among people living with HIV who had initiated ART in rural Uganda. Age and texting experience were also associated with high behavioral intention to use the texting system. These findings highlight the key ingredients of acceptable mHealth interventions in this population. Our study also suggests the need for longitudinal technology acceptability data among people living with HIV in resource-limited settings to better understand how acceptability changes over time among patients for whom long-term engagement in care is paramount.

## Appendix

Multimedia Appendix 1 Exploratory factor analysis loadings for items measuring Unified Theory of Acceptance and Use of Technology constructs.

Multimedia Appendix 2 Marginal effects of social influence, performance expectancy, and effort expectancy on the probability of high behavioral intention.

Multimedia Appendix 3 Marginal effects of covariates on the probability of high behavioral intention.

Multimedia Appendix 4 Results of sensitivity analyses. Association of covariates with behavioral intention, with no Unified Theory of Acceptance and Use of Technology (UTAUT) construct (column 1), and association of each UTAUT construct adjusting for covariates but not other UTAUT constructs (columns 2-9). Effect estimates and confidence intervals for covariates are shown only once, and did not vary substantially.

## Abbreviations

aOR: adjusted odds ratio
ART: antiretroviral therapy
EFA: exploratory factor analysis
mHealth: mobile health
UTAUT: Unified Theory of Acceptance and Use of Technology
