# Supplementary material for: Behavioral Predictors of Intention to Use a Text Messaging Reminder System Among People Living With HIV in Rural Uganda: Survey Study
Source: JMIR Hum Factors. 2023 May 5;10:e42952. doi: 10.2196/42952 (PMC10199388; doi:10.2196/42952)
Supplement: Multimedia Appendix 1 [file humanfactors_v10i1e42952_app1.docx]

**Supplemental table 1.** Exploratory factor analysis loadings for items measuring UTAUT constructs.

| **Summary of survey items and constructs** | **Loading in EFA** |
| --- | --- |
| *Performance expectancy* |  |
| PE1. I would find the SMS program useful | 0.770 |
| PE2. Using the SMS program enables me to get care from the clinic more quickly. | 0.907 |
| PE3. Using the SMS program increases my ability to get help from the clinic. | 0.981 |
| PE4. If I use the SMS program, I will increase my chances of getting help at the clinic. | 0.984 |
| *Effort expectancy* |  |
| EE1. My interaction with the SMS program would be clear and understandable. | 0.989 |
| EE2. It would be easy for me to become skillful at using the SMS program. | 0.984 |
| EE3. I would find the SMS program easy to use. | 0.991 |
| EE4. Learning to operate the SMS program is easy for me. | 0.984 |
| *Attitude toward using technology* |  |
| ATT1. Using the SMS program is a good idea. | 0.470 |
| ATT2. The SMS program makes work more interesting. | 0.836 |
| ATT3. Working with the SMS program is fun. | 0.955 |
| ATT4. I like working with the SMS program. | 0.909 |
| *Social influence* |  |
| SI1. People who influence my behavior think that I should use the SMS program. | 0.518 |
| SI2. People who are important to me think that I should use the SMS program. | 0.527 |
| SI3. The clinic staff have been helpful in the use of the SMS program. | 0.935 |
| SI4. In general, the clinic has supported the use of the SMS program. | 0.943 |
| *Facilitating conditions* |  |
| FC1. I have the resources necessary to use the SMS program. | 0.505 |
| FC2. I have the knowledge necessary to use the SMS program. | 0.793 |
| FC3. The SMS program is not compatible with other SMS programs I use. | 0.785 |
| FC4. A specific person (or group) is available for assistance with SMS program difficulties. | -0.489 |
| *Self-efficacy:* I could successfully get the information I need using the SMS program… |  |
| SE1. If there was no one around to tell me what to do as I go. | 0.705 |
| SE2. If I could call someone for help if I got stuck. | -0.159 |
| SE3. If I had a lot of time, I could get the information I needed from the SMS program. | 1.1690 |
| SE4. If I had just the help of the information I received at the clinic about the SMS program | 0.484 |
| *Anxiety* |  |
| ANX1. I feel apprehensive about using the SMS program. | 0.893 |
| ANX2. It scares me to think that I could lose a lot of information using the SMS program by hitting the wrong key | 0.869 |
| ANX3. I hesitate to use the SMS program for fear of making mistakes I cannot correct. | 0.857 |
| ANX4. The SMS program is somewhat intimidating to me. | 0.910 |
| *Behavioral intention to use* |  |
| BI1. I intend to use the SMS program in the next 3 months. | 0.985 |
| BI2. I predict I would use the SMS program in the next 3 months. | 1.015 |
| BI3. I plan to use the SMS program in the next 3 months. | 0.985 |

Abbreviations: EFA – exploratory factor analysis; SMS – short message service; UTAUT - Unified Theory of Acceptance and Use of Technology
