# Supplementary figures and images for: Behavioral Predictors of Intention to Use a Text Messaging Reminder System Among People Living With HIV in Rural Uganda: Survey Study
Source: JMIR Hum Factors. 2023 May 5;10:e42952. doi: 10.2196/42952 (PMC10199388; doi:10.2196/42952)

## Slide 1
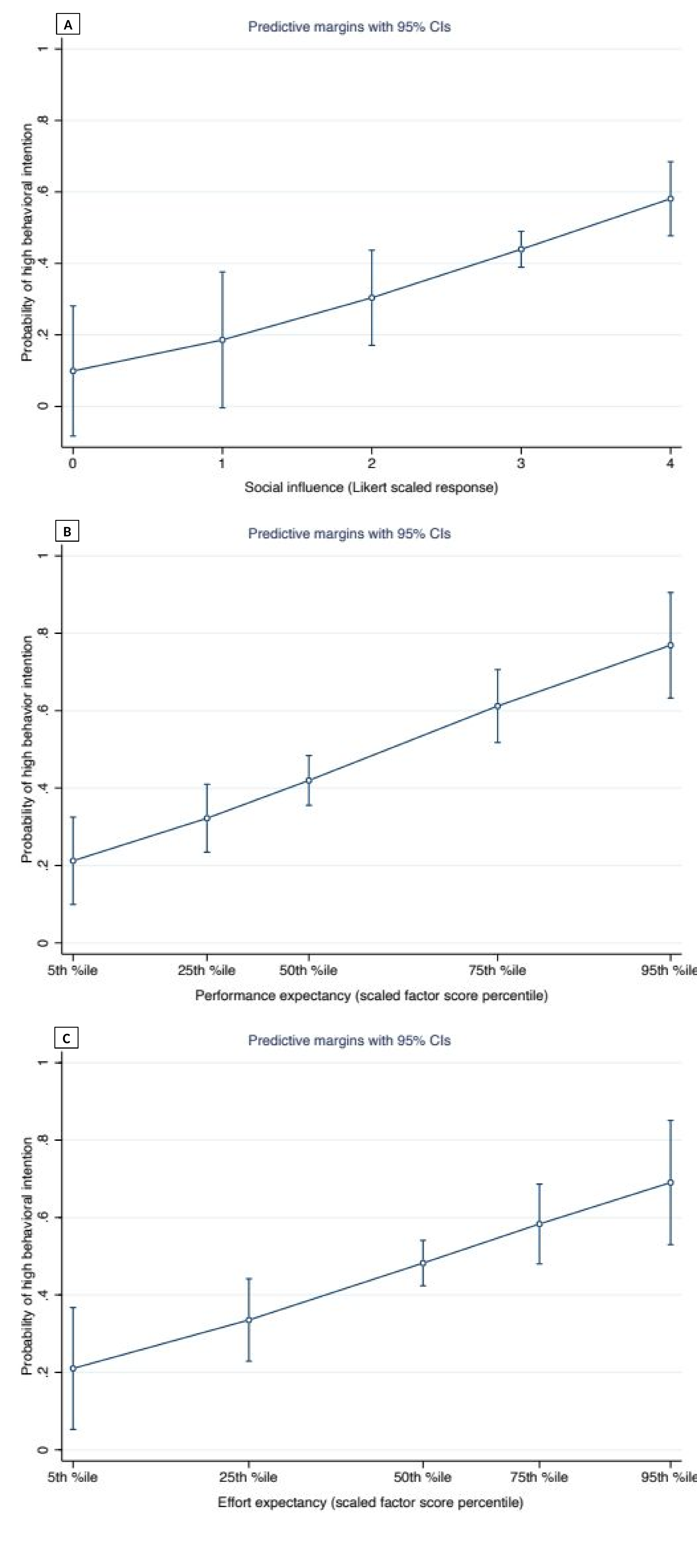

A
B
C

Supplement: Multimedia Appendix 2 [file humanfactors_v10i1e42952_app2.pptx]

## Slide 1
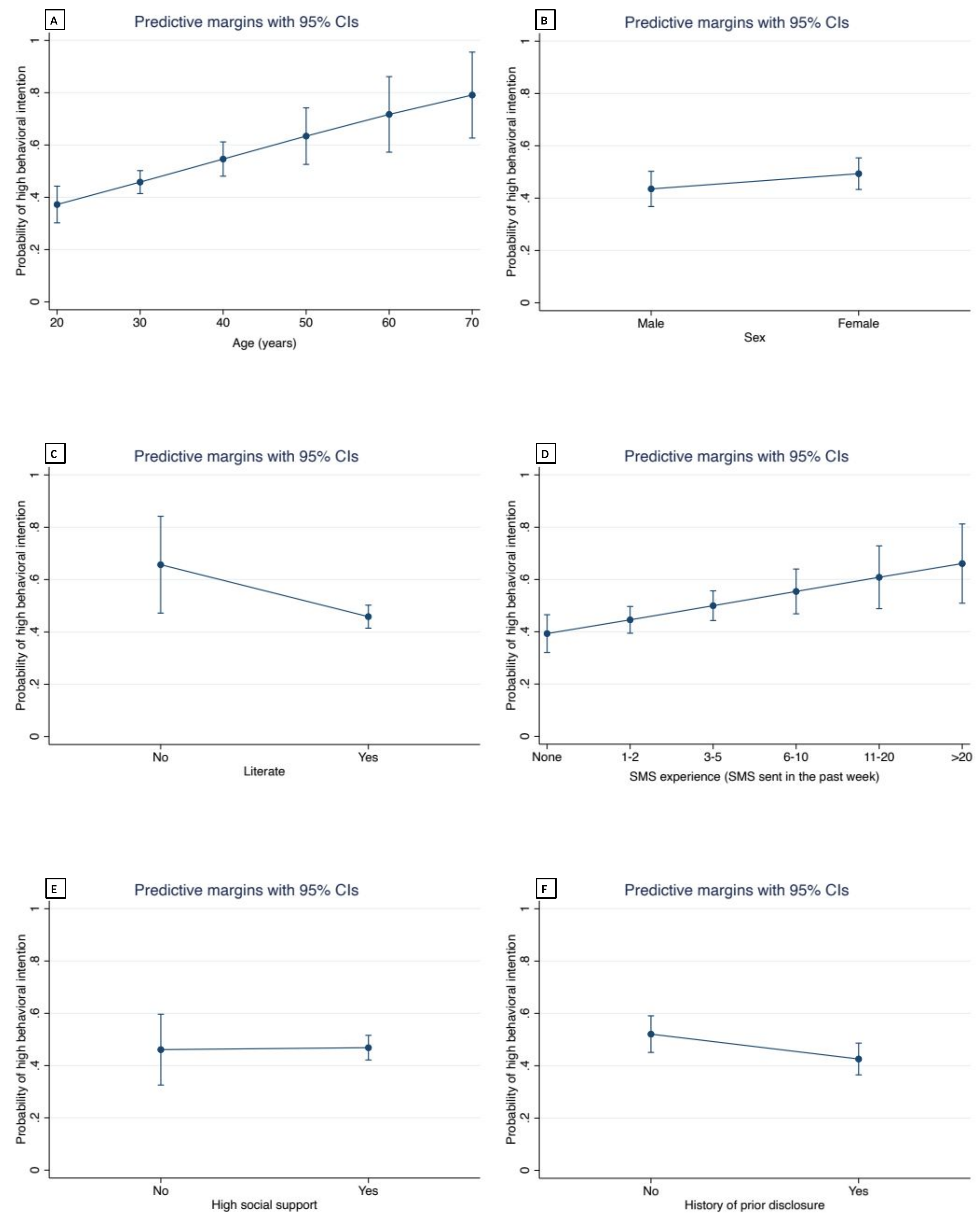

A
B
C
D
E
F

Supplement: Multimedia Appendix 3 [file humanfactors_v10i1e42952_app3.pptx]
