# Supplementary material for: Behavioral Predictors of Intention to Use a Text Messaging Reminder System Among People Living With HIV in Rural Uganda: Survey Study
Source: JMIR Hum Factors. 2023 May 5;10:e42952. doi: 10.2196/42952 (PMC10199388; doi:10.2196/42952)
Supplement: Multimedia Appendix 4 [file humanfactors_v10i1e42952_app4.docx]

**Supplemental table 2.** Results of sensitivity analyses. Association of covariates with behavioral intention, with no UTAUT construct (column 1), and association of each UTAUT construct adjusting for covariates but not other UTAUT constructs (columns 2-9). Effect estimates and confidence intervals for covariates are shown only once, and did not vary substantially

| Multivariable model | All UTAUT constructs^1^ + age + sex | All UTAUT constructs^1^ + all covariates^2^ | PE + all covariates^2^ | EE + all covariates^2^ | ATT + all covariates^2^ | FC + all covariates^2^ | ANX + all covariates^2^ | SE + all covariates^2^ | SI-1 + all covariates^2^ | SI-2 + all covariates^2^ |
| --- | --- | --- | --- | --- | --- | --- | --- | --- | --- | --- |
| Performance expectancy (PE), OR (95%CI)^3^ | **6.40 (2.80-14.66)** | **6.78 (2.91-15.80)** | **10.52**  **(5.41-20.47)^1^** |  |  |  |  |  |  |  |
| Effort expectancy (EE), OR (95%CI)^3^ | 6.95 (0.26-182.88) | 8.87 (0.26-296.92) |  | **16.93**  **(6.91-41.49)^1^** |  |  |  |  |  |  |
| Attitudes (ATT), OR (95%CI)^3^ | 0.82 (0.15-4.64) | 0.49 (0.08-3.03) |  |  | **10.60**  **(4.86-23.10)^1^** |  |  |  |  |  |
| Facilitating conditions (FC), OR (95%CI)^3^ | 0.85 (0.05-14.17) | 0.62 (0.03-13.25) |  |  |  | **12.56**  **(5.51-28.65)^1^** |  |  |  |  |
| Anxiety (ANX), OR (95%CI)^3^ | 1.64 (0.65-4.13) | 1.70 (0.63-4.55) |  |  |  |  | **0.33**  **(0.19-0.57)^1^** |  |  |  |
| Self-efficacy (SE), OR (95%CI)^3^ | 1.38 (0.18-10.44) | 2.19 (0.25-19.20) |  |  |  |  |  | **9.75  (4.53-20.95)^1^** |  |  |
| Social influence (“The clinic staff have been helpful in the use of the SMS program”) (SI-1), OR (95%CI)^4^ | **2.53 (1.03-6.24)** | **3.20 (1.23-8.38)** |  |  |  |  |  |  | **4.48**  **(2.17-9.23)^2^** |  |
| Social influence (“People who are important to me think that I should use the SMS program”) (SI-2), OR (95%CI)^4^ | 1.11 (0.67-1.84) | 1.06 (0.63-1.81) |  |  |  |  |  |  |  | **1.93**  **(1.33-2.80)^2^** |

^1^UTAUT constructs are performance expectancy, effort expectancy, attitudes, facilitating conditions, anxiety, self-efficacy, and social influence (left-hand column)

^2^Covariates are age, sex, literacy, SMS experience, social support, and HIV status disclosure.

^3^Odds ratio associated with a 1 unit change in the factor score interquartile interval

^4^Odds ratio associated with a 1-unit change in the Likert scale

Abbreviations: PE – performance expectancy; EE – effort expectancy; ATT – attitudes; FC – facilitating conditions; ANX – anxiety; SE – self-efficacy; SI-1 – self efficacy (first question); SI-2 – self efficacy (second question)
